# Association of Serum Hepcidin Levels with Aerobic and Resistance Exercise: A Systematic Review

**DOI:** 10.3390/nu13020393

**Published:** 2021-01-27

**Authors:** Phureephat Larsuphrom, Gladys Oluyemisi Latunde-Dada

**Affiliations:** Department of Nutritional Sciences, School of Life Course Sciences, King’s College London, Franklin-Wilkins-Building, 150 Stamford Street, London SE1 9NH, UK; phureephat.larsuphrom@kcl.ac.uk

**Keywords:** hepcidin, resistance, aerobic, IL-6, exercise, anaemia

## Abstract

Background: Prevalence of iron deficiency is commonly reported among athletic population groups. It impairs physical performance due to insufficient oxygen delivery to target organs and low energy production. This is due to the high demand of exercise on oxygen delivery for systemic metabolism by the erythrocytes in the blood. Hepcidin, the key regulator of iron homeostasis, decreases to facilitate iron efflux into the circulation during enhanced erythropoiesis. However, acute anaemia of exercise is caused by increased hepcidin expression that is induced by stress and inflammatory signal. The study aimed to systematically review changes in serum hepcidin levels during resistance and aerobic exercise programmes. Methods: A systemic literature search from 2010 to April 2020 across seven databases comprised of Cochrane library, PubMed, Web of Science, Scopus, Embase, MEDLINE, and OpenGrey. The primary outcome was increased or decreased serum hepcidin from baseline after the exercise activity. Risks of bias were evaluated by using the National Institutes of Health (NIH) for quality assessment of before and after different exercise programmes. Results: Overall, twenty-three studies met the inclusion criteria. Out of the 23 studies, 16 studies reported significantly exercise-induced serum hepcidin elevation. Of the 17 studies that evaluated serum interleukin (IL)-6 levels, 14 studies showed significant exercise-induced serum IL-6 elevation. Changes in exercise-induced serum hepcidin and IL-6 levels were similar in both resistance and endurance exercise. Significant correlations were observed between post-exercise hepcidin and baseline ferritin levels (*r* = 0.69, *p* < 0.05) and between post-exercise hepcidin and post-exercise IL-6 (*r* = 0.625, *p* < 0.05). Conclusion: Resistance and endurance training showed significant increase in serum hepcidin and IL-6 levels in response to exercise. Baseline ferritin and post-exercise IL-6 elevation are key determining factors in the augmentation of hepcidin response to exercise.

## 1. Introduction

### 1.1. Background

Iron is an essential mineral for a number of physiological processes, and it is crucial for health and physical performance. It plays an essential role in the human body because it is involved in the production of oxygen-carrying proteins, haemoglobin, and myoglobin, which deliver oxygen to target tissues. Iron is also involved in energy formation within the electron transport chain, DNA synthesis, and in oxidative phosphorylation in the mitochondria [1]. These functions make iron an essential element that is relevant to physical performance. Therefore, iron deficiency (ID) leads to impaired energy metabolism due to reduced cytochrome production. This results in impaired ATP production and energy metabolism that contributes to detrimental exercise performance [2,3]. Iron deficiency also causes fatigue, exercise intolerance, impaired immune function and temperature regulation, and impairs the capability to adapt to high altitudes and stress [4,5]. Hence, an optimal iron status is required to maintain good exercise performance throughout the sports season.

### 1.2. Epidemiology of Iron Deficiency in Athletes

ID is a common nutritional condition worldwide, and the number of cases with or without anaemia is 16% to 57% of female athletes and 1% to 31% of male athletes [6]. Although a high intake of iron is often promoted in athletes, the prevalence of ID is relatively high in athletes because iron absorption is negatively affected by physical exercise [7]. Haemoglobin and ferritin levels are lower in athletes compared to non-athletes [6]. The incidence of iron depletion in athletes who participate in endurance sports has been reported to be between 30% and 50%, and this is due to gastrointestinal bleeding, haemolysis, haematuria, and inadequate iron intake [8]. High risk of iron depletion is observed in female athletes due to inadequate iron intake and blood loss during menstruation [9]. Athletes who are vegetarians are also at a high risk of ID due to low iron in plant-based foods that contain high levels of inhibitors such as phytate, fibre, and polyphenols. Dietary iron is absorbed when duodenal cytochrome B (DcytB) converts Fe^3+^ into Fe^2+^ in the intestinal lumen [10]. Fe^2+^ is transported across the cell membrane by the divalent metal transporter 1 (DMT1). Inside the cell, iron is stored as ferritin or transported out of the cell by ferroportin (FPN) into the blood circulation [11]. A study reported that a decrease in duodenal DMT1 and FPN was observed in rats after vigorous exercise compared to the controls [12]. This response causes reduced iron absorption, which compromises iron status in athletes [13].

### 1.3. Hepcidin and Iron Metabolism

Hepcidin is a peptide hormone that is produced by hepatocytes. It is known as a master regulator of iron homeostasis as it inhibits iron efflux from the duodenum, macrophages, and the placenta [14,15]. Hepcidin enhances the degradation and ubiquitination of ferroportin to inhibit iron release from the enterocyte into blood circulation. Hepcidin expression is regulated by iron status, erythropoiesis, inflammation, and hypoxia. Stress conditions such as anaemia and hypoxia promote erythropoiesis, which decreases hepcidin activity [16]. Suppression of hepcidin enhances increased iron entry into the plasma to meet the required high demand for iron delivery to the bone marrow for erythropoiesis. Some endurance athletes commonly undertake an exercise programme in hypoxic environments to improve performance because of increased red blood cell volume [17]. A hypoxic condition stimulates the kidney to produce erythropoietin (EPO), which promotes the proliferation of erythroid precursor cells. Increased EPO production by the kidney stimulates erythroferrone (ERFE) production. ERFE is a hormone that is produced by erythroblasts to inhibit hepcidin expression [18]. To induce hepcidin expression, ERFE binds to BMP6 and BMP6/BMP2 heterodimers to block BMP/SMAD signalling [19].

### 1.4. Hepcidin, Interleukin-6, and Exercise

Under inflammatory conditions, increased serum interleukin-6 (IL-6) activates hepcidin expression via JAK/STAT3 signalling cascades, resulting in the promotion of hepcidin transcription [20]. Exercise and physical activities increase physiological demand and promote inflammation, which influences hepcidin expression and results in the development of acute anaemia of exercise (AAE). Iron metabolism during AAE and the regulation of hepcidin expression are reviewed in [21]. This increase is magnified by increased IL-6 levels in response to exercise [22].

Changes in urinary hepcidin were first reported in women who had completed a marathon [23]. Most studies reported that a twofold to fourfold increase in serum hepcidin was detected following acute bouts of exercise at an intensity between 60% and 90% of VO_2max_, peaking at 3 h after exercise [24,25,26,27]. The magnitude of these responses was mediated by baseline iron status and IL-6 [28,29]. By contrast, there was no significant difference in serum and urinary hepcidin levels in humans after submaximal cycling exercise [30,31].

Although there is a growing body of evidence on the subject, the relevance of hepcidin’s response to exercise and iron deficiency anaemia has not sufficiently provided clear results to draw a firm conclusion. Moreover, most of the published studies investigated aerobic exercises, except a recent one that reported on resistance exercise. While resistance and aerobic exercise programmes are both important to professional athletes, they are also embraced by diverse groups in the population for long-term health benefits. Therefore, a comprehensive understanding of the changes in serum hepcidin in response to resistance and aerobic exercise will inform athletes and clinicians on the consequence of iron-deficiency anaemia and its management. The systematic review investigated changes in serum hepcidin levels during resistance and aerobic exercise programmes. Additionally, it aimed to evaluate the effect of exercise on serum IL-6 in athletes or recreationally trained subjects.

## 2. Methods

This systematic review was conducted in accordance with the Preferred Reporting Items for Systematic Reviews and Meta-Analyses guidelines 2009 (PRISMA 2009) [32]. Registration number: CRD42020188203.

### 2.1. Eligibility Criteria

The PICOS criteria are shown in Table 1. The inclusion criteria were as follows: (a) experimental studies that examined the effect of exercise on serum hepcidin and IL-6 levels that were determined by valid methods, (b) studies conducted with athletes or recreationally trained participants, and (c) studies published in the English language. Systematic reviews, meta-analyses, surveys, abstract, and conference reports were excluded, as were studies conducted with animal models. Finally, studies that did not measure serum hepcidin levels or did not provide sufficient information to evaluate changes in serum hepcidin were excluded.

### 2.2. Search Strategy and Identification of Studies

The literature search was conducted across seven databases (Cochrane library, PubMed, Web of Science, Scopus, Embase, MEDLINE, and OpenGrey for unpublished literature) to select relevant experimental studies. The search strategy combined the following Medical Subject Heading (MeSH) and Embase thesaurus: Aerobic AND Resistance AND Exercise AND Hepcidin AND Experimental AND Urinary were the keywords used to search for the relevant studies. Subsequently, Aerobic OR Resistance OR Exercise OR Hepcidin OR Experimental studies OR Urinary hepcidin were also used to search for relevant studies. Finally, synonyms such as Cardiovascular OR Aerobic OR Workout OR Training OR Endurance OR Resistance were used to search for relevant studies.

Initially, obvious irrelevant studies were excluded after screening titles and abstracts. The full texts of the other remaining articles were carefully evaluated according to the eligibility criteria. Where required, and when in doubt, the eligibility for inclusion was resolved by two independent individuals (P.L.) and (G.O.L.D.).

### 2.3. Data Extraction

Data were extracted independently by two reviewers based on the following: name of the authors, publication year, country, number of participants, the proportion of female participants, mean age/age range of participants, population description, subject demographics, study setting, baseline characteristics, details of the intervention, study methodology, inclusion and exclusion criteria, recruitment, dropout rate, outcomes, and times of measurement information for the assessment of the risk of bias. The main findings, relevant results, and *p*-values were also reported. The second author reviewed the extracted data and both authors agreed on individual items that were vague and doubtful.

### 2.4. Strategy for Data Synthesis

We provide a narrative synthesis of the findings from the included studies based on the PICOS criteria. We also provide a narrative synthesis structured on the type of exercise intervention. For example, we compared different types of exercises (resistance vs. aerobic exercise) and different intensities of exercise (moderate vs. high intensity of aerobic exercise). The target population characteristics were analysed by considering sample size, gender, and age in order to assess the designs and exercise programmes before comparing each study. Continuous data were selected for the review so as to compare studies and reported data on the increase or decrease in serum hepcidin levels from baseline values. Mean differences in serum hepcidin responses were compared. Moreover, we assessed the validity of the methods used to analyse serum hepcidin levels to ensure the reliability and quality of the data in the studies included in the review.

### 2.5. Assessment of Risk of Bias

Two reviewers (P.L. and G.O.L.D.) used the quality assessment tools of the National Institutes of Health (NIH) for quality assessment of before-and-after studies without control groups. Two reviewers scored the 23 studies in this review across 12 questions in order to assess the internal validity of each study and, thus, ensure the overall high standard of each study. Depending on the study design, each of the 12 questions was answered with either “yes”, “no”, or “other cannot determine (CD), not reported (NR), not applicable (NA)”. This allowed determination of the rating of each study as either “good”, “fair”, or “poor”.

### 2.6. Outcomes

#### 2.6.1. Primary Outcome

Changes in serum hepcidin levels from baseline to the last exercise training protocol. Measures of effect are an increase or decrease in these from baseline values.

#### 2.6.2. Secondary Outcome

Changes in serum IL-6 levels from baseline to the last exercise training protocol. Measures of effect are an increase or decrease in these from baseline values.

### 2.7. Analysis

Serum hepcidin, urinary hepcidin, and IL-6 were converted to conventional units in order to compare them across all studies, i.e., ng/mL, nM/mmol Cr, and pg/mL, respectively.

## 3. Results

### 3.1. Identification and Selection of the Included Articles

**Figure 1:**
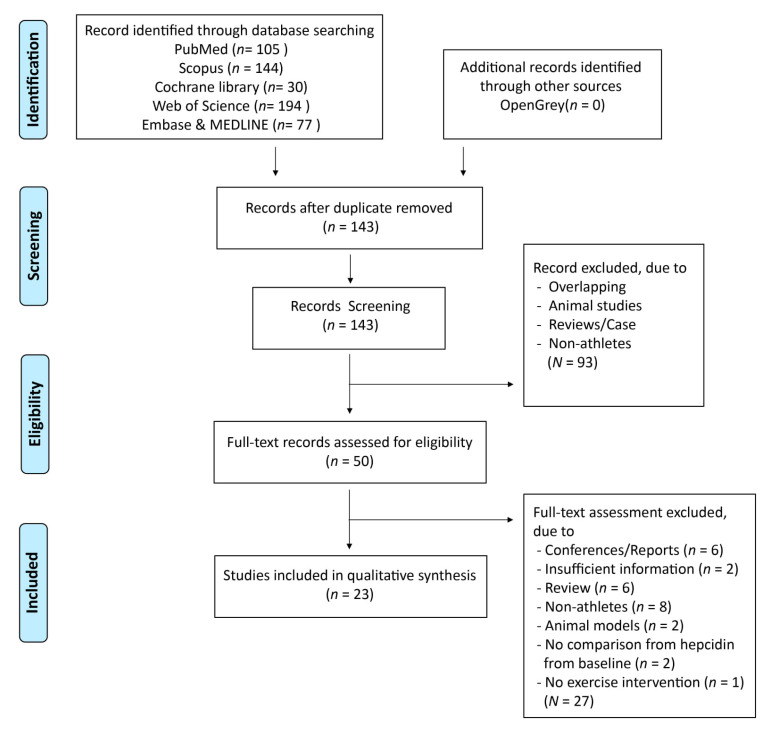
Flow diagram of selection process.

A summary of the study selection process is shown in Figure 1. A total of 550 articles were identified through seven databases (Cochrane Library, PubMed, Web of Science, Scopus, Embase, MEDLINE, and OpenGrey) since 2010. After duplicates were removed, 143 articles were further screened. Of these, 93 articles were excluded after assessing the title and abstracts, and the reasons for exclusion are shown in Figure 1. Fifty articles were evaluated for eligibility after reading the full-texts. A total of 27 articles were excluded because they were conference reports, lacked qualitative data, were selective bias reports, or had non-athletes as subjects (Figure 1).

### 3.2. Description and Characteristics of the Studies

#### 3.2.1. Participant Characteristics

The 23 studies included in the current review (Table 2) comprised 376 participants (20 recreational participants and 356 athletes). Few studies had more than 20 participants, while most studies involved less than 20 participants. The average age of study participants ranged from 17.8 to 39.29 years, and the majority of the participants were men (76.5% of all participants). Regarding geographic distribution, eleven studies were conducted in Europe (Poland, *n* = 5; Slovenia, *n* = 2; Germany, *n* = 2; Spain, *n* = 1; Switzerland, *n* = 1). Eleven studies were conducted in Asia (Australia, *n* = 8; Japan, *n* = 3), while only one study was conducted in the USA (Table 2).

#### 3.2.2. Study Design and Methods Used

Of the 23 studies, 12 were single-arm pilot studies and 11 were cross-over designs published in the English language. Most of the studies conducted involved athletes, while only two studies recruited recreationally trained participants. In terms of analysis of serum hepcidin and urinary hepcidin, 14 studies performed analyses using enzyme-linked immunosorbent assay (ELISA), while nine studies performed analyses using a combination of weak cation exchange chromatography and time-of-flight mass spectrometry (WCX-TOF MS). A total of 17 studies determined both serum hepcidin and IL-6 levels, while six studies analysed only serum hepcidin.

Of the 23 selected studies, 22 were conducted during aerobic exercise training. Interestingly, the first study that compared resistance exercise to aerobic exercise and controls is included in the present review. Regarding the 22 studies that employed aerobic exercise, nine of them included non-weight-bearing exercises such as cycling and rowing, while the rest of the studies included weight-bearing exercises such as running, sprinting, or walking exercises. A total of 15 studies investigated high-intensity exercise as an intervention, while three studies were conducted during moderate-intensity exercise. Three studies evaluated both moderate- and high-intensity exercise. Six studies investigated the cumulative effect of exercise on serum hepcidin, while 17 of 23 studies assessed the effect of acute bouts of exercise on serum hepcidin.

### 3.3. Outcome

The main findings of studies that analysed serum hepcidin in response to different exercise protocols are summarised in Table 3.

#### 3.3.1. Serum Hepcidin and IL-6 Levels

Most of the 16 studies revealed that exercise resulted in significantly increased serum hepcidin levels, while four studies reported significantly decreased serum hepcidin levels after exercise. Three studies demonstrated no significant changes in serum hepcidin levels after exercise. Out of 17 studies that evaluated serum IL-6, 14 reported significant exercise-induced increases in serum IL-6 levels, and three studies reported no change in serum IL-6 levels after exercise.

#### 3.3.2. Risk of Bias within Studies

Each study was assessed using the NIH Quality Assessment Tool for before-and-after (Pre–Post) studies without control groups (Supplementary Data). The majority of the studies had a “fair” risk of bias. The risk of bias of six studies was graded as “good”. Five studies were graded as having a “poor” risk of bias.

Of the 23 studies included, 20 reported the eligibility criteria. Only five of the studies reported the sample size based on power and a significance level of 0.05. All studies used statistical methods to set *p*-values for the pre-to-post changes. A study dropout of more than 20% was observed in one study [34]. Most studies described clearly defined interventions regarding intensity and the exercise protocol, while two studies lacked exercise intensity specifications.

## 4. Discussion

### 4.1. Summary of Evidence

Exercise performance has been associated with elevated levels of serum hepcidin and IL-6 in athletes. This review revealed that the majority of the studies confirmed a significant increase in serum hepcidin (16 of 23 studies) and serum IL-6 levels (14 of 17 studies) in response to exercise. In general, a peak in hepcidin elevation was observed at 3 h after exercise [52]. Out of 16 studies that reported increased serum hepcidin in the current review, 11 showed increased hepcidin, with a peak at 3 h after exercise. However, others demonstrated the peak of hepcidin levels immediately or at 1 or 2 h after exercise [38,47,51] instead. These latter studies that reported serum hepcidin elevations with a peak before 3 h after exercise excluded the 3-h time point, and the data showed increasing serum hepcidin over the time-course of the training exercise. The outcome of the current systematic review revealed that serum hepcidin’s response to moderate- to high-intensity exercise occurs rapidly and reaches a peak at 3 h, returning to baseline levels at 6 h after exercise. Different types of exercise modalities, either resistance or endurance training, did not differ in the 3-h peak in post-exercise serum hepcidin levels [33].

Exercise intensity reported in the current review comprises moderate and high intensities. The study reported elevated hepcidin concentration after high-intensity endurance training, reaching a peak at 3 h after exercise, similar to moderate intensity. However, there was no significant difference in the effect size of exercise-induced serum hepcidin elevation between high-intensity (85% VO_2max_) and moderate-intensity exercise (65% VO_2max_) [25]. The current review found a similar elevation in serum hepcidin in response to different exercise intensities from 60% to 90% of VO_2max_, thus indicating that exercise intensity may not be vital in the response of hepcidin to endurance training. However, the effects of low-intensity exercise on hepcidin remain unknown.

Apart from exercise intensity, training volume is proposed as an important factor in post-exercise hepcidin elevation. Although the current study showed a significant increase in serum hepcidin following 120 min vs. 60 min endurance exercise at the same intensity (60% of VO_2max_), a high training volume resulted in greater hepcidin levels than a low training volume (60 min) [26]. This result is in contrast to the result of a previous study that investigated a 40-min vs. 90-min endurance exercise at 75% VO_2max_ and demonstrated that there was no significant difference in change in hepcidin levels between trials [44]. The contrasting findings may be due to a significantly higher fatigue level at 120 min as a result of reduced muscle glycogen, leading to a different hepcidin release [53]. However, the fatigue-dependent mechanism was not supported by Kasprowic et al. [50], who evaluated the hepcidin response during and after a 100-km run (~10 h long). The authors reported that despite the longer time and higher volume, there was no significant difference in serum hepcidin levels between exercise durations. Based on the current finding, training volume does not seem to be a vital factor in the response of hepcidin to endurance training.

Although there is a growing body of evidence regarding the fact that endurance exercise causes increased serum hepcidin levels, that of the effect of resistance training on serum hepcidin levels is scarce. A study on rats that compared resistance training to aerobic exercise showed an increase in haemoglobin concentration, without an impact on iron status, as well as an enhancement of iron absorption as a result of increased recycled iron [54]. The research demonstrated that energy expenditure during workouts is inversely correlated with increased hepcidin levels after exercise [55]. Resistance training is thought to be unlikely to stimulate hepcidin because of the lower energy that is expended. However, some other reports suggested that resistance training might induce inflammation due to reduced muscle glycogen and increased IL-6 expression [56,57]. Recently, Goto et al. [33] investigated the effect of resistance exercise and aerobic exercise on IL-6 and hepcidin 6 h after exercise in 10 recreationally trained males. The study found that a single bout of resistance exercise (RE) and endurance training (ENT) significantly elevated serum hepcidin, with a more significant increase in RE than in ENT at 3 h. Serum IL-6 levels, however, increased immediately from baseline levels with no significant difference between the trials. This finding indicated that a greater increase in serum hepcidin levels after RT than after ENT was not augmented by IL-6.

Haemolysis has been linked to a mechanical impact on the body in some types of sports such as running and walking. Research shows that higher degrees of haemolysis were observed after intensity-matched running compared to a cycling session at 75% VO_2max_, indicating that impact forces influence the rate of haemolysis [58]. Moreover, a study reported significantly increased levels of hepcidin in four separate exercise protocols in 10 triathletes that comprised cycling at moderate- and high-intensity and running at high- and moderate-intensity regimes. The lowest magnitude of the effect was observed in moderate-intensity cycling compared to other trials, suggesting that athletes who are at risk of iron deficiency should perform moderate-intensity, non-weight bearing exercise [25]. However, some studies revealed that exercise-induced hepcidin elevation might occur in response to haemolysis, and it is not augmented by mechanical impact. Studies confirmed this assumption and found that different training surfaces did not influence serum hepcidin, inflammatory markers, and haemolysis in athletes [27,44]. Interestingly, Goto et al. [33] demonstrated that post-exercise serum iron elevation as an indirect marker of haemolysis was 10% higher in resistance exercise than in cycling. The increased iron loss from haemolysis can be a major factor that promotes hepcidin biosynthesis [59]. However, there are no studies that have evaluated the effect of resistance training on hepcidin compared to weight-bearing exercise (running). Therefore, future studies need to be conducted before concrete conclusions are drawn.

Hepcidin responses are sensitive to acute exercise bouts. However, studies that investigated cumulative effects of exercise are still scarce. Auersperger et al. [49] evaluated the effect of an 8-week endurance training programme on serum hepcidin levels and reported a decrease in serum hepcidin 3 weeks after training load. This was presumably caused by the increased requirement of iron in athletes as an adaptation process [13]. Furthermore, Auersperger et al. [48] reported that serum hepcidin decreased during an eight-week training programme regardless of the baseline iron status, and there was no significant change in C-reactive protein (CRP), indicating that the decreased serum hepcidin was associated with homeostatic regulation due to high iron demand rather than being a consequence of inflammation. This finding aligns with Moretti et al. [40] who reported decreased hepcidin and increased EPO after 3 weeks of an endurance exercise programme in recreational males as a consequence of erythropoietic drive influenced by exercise. Judging by the prevailing evidence, acute exercise seems to promote post-exercise serum hepcidin elevation, while long-term exercise training might attenuate exercise-induced increased hepcidin.

Another factor that accounts for variations in hepcidin response to exercise is the time of day when the activity is performed. A study evaluated the effect of morning versus afternoon exercise, i.e., a 90-min running protocol on iron absorption, including serum hepcidin and IL-6 levels. A twofold increase in serum hepcidin was observed in the afternoon trial, while a threefold increase was observed in the morning trial [35]. However, increased iron absorption was observed at breakfast after morning trials. This phenomenon can be explained either by a combination of a diurnal variation over the whole day or by some mechanisms that promote high iron absorption in the morning with a higher increase in serum hepcidin than that in the afternoon trial. These mechanisms require further investigation. In the same study, endurance training caused a significant twofold to fourfold increase in serum IL-6 levels at 3 h after exercise. This increase persisted throughout the day after the morning endurance training. These results agree with those of a previous study that reported a threefold to fivefold increase in serum IL-6 between the hours of 8:00 and 19:00 [60].

### 4.2. Mechanism of Exercise-Induced Hepcidin and IL-6 Expression

Baseline ferritin levels or iron status could determine hepcidin response to exercise. A study found that increased serum hepcidin was observed only in athletes with baseline ferritin levels above 30 μg/mL [45,52], indicating that low iron stores attenuate post-exercise hepcidin elevation. The correlation between hepcidin and iron parameters was reported by Peeling et al. [29], who found a significant positive correlation between post-exercise hepcidin levels and baseline ferritin levels (r = 0.69, *p* < 0.01) [41]. A further study by Burden [61] showed that iron supplementation promoted increased hepcidin elevation after aerobic exercise in iron-deficient athletes. In the current systematic review, most of the studies reported post-exercise hepcidin elevation when the ferritin level was above 30 μg/mL. Consequently, these studies indicate that the baseline iron status of athletes plays an important role in hepcidin’ response to endurance exercise.

Exercise leads to increased physiological demand of energy, promotes inflammation, and increases IL-6 expression, which results in enhanced hepcidin expression [13]. This association was confirmed by Peeling et al. [29], who reported a significant correlation between 3 h post-exercise hepcidin and post-exercise serum IL-6 levels (r = 0.625, *p* < 0.05). This result was also in line with Banzet et al. [22]. The effect of exercise on serum IL-6 levels depends on the intensity and duration of the exercise. Sim et al. [25] reported a significant difference in serum IL-6 between high- and moderate-intensity running, while there was no difference between weight-bearing exercise (run trial) and non-weight-bearing exercise (cycle trial). This difference in the serum IL-6 response to different intensity exercises is in line with the observation made by Peeling et al. [27]. Moreover, repeated running exercise with intensity above 90% VO_2max_ contributed to a significantly higher inflammatory response (IL-6) than that at the intensity of 75–80% VO_2max_, resulting in a more considerable increase in hepcidin levels [62]. However, the effect of exercise on IL-6 was not affected by training volume and exercise modality [26]. In summary, the present review found that 15 of the 17 studies that measured serum IL-6 levels showed significant post-exercise IL-6 elevation, reaching peak values immediately after exercise.

Hypoxia is another factor known as a negative regulator of hepcidin through hypoxia-inducible factor 1α (HIF-1α) [63]. Consistent with this concept, deletion of the von Hippel–Lindau factor (VHL) gene that controls HIF-1α in the liver cell caused decreased hepcidin levels and increased ferroportin levels [64]. In a human trial, Badenhorst et al. [46] found a significant decrease in hepcidin concentration at 3 h post-exercise under hypoxic conditions. By contrast, Govus et al. [41,43] reported a significant increase in hepcidin levels at 3 h post-exercise under hypoxic conditions, similar to the normoxic condition. Additionally, the author found that the magnitude of response of serum hepcidin to exercise under normoxic or hypoxic conditions is dependent on baseline ferritin levels. This result is consistent with the result of the study by Peeling et al. [29], who reported that baseline ferritin and iron levels could explain 77% of post-exercise hepcidin elevation. Therefore, baseline ferritin levels play a predominant role in increased hepcidin in response to exercise. However, the inconsistent results may be due to exposure time to the hypoxic condition, and a small increase in EPO after exercise under a hypoxic condition might not be enough to attenuate hepcidin synthesis.

Increased serum hepcidin in response to exercise, apart from the duodenum, also inhibits iron efflux from hepatocytes and splenic macrophages [59]. A study reported significant decreases in indicators of iron status (iron, ferritin, and total iron-binding capacity (TIBC)) following aerobic exercise [52], resulting in reduced iron into the bone morrow. Insufficient serum iron in the bone marrow decreases erythropoiesis, which results in an anaemic condition. Notably, Reinke et al. [65] reported that 27% of absolute iron deficiency was observed in senior elite rowers at the end of the competitive season. This was in line with the findings by Auersperger et al. [48], who reported that a decreased iron store by 71% was observed in runners at the end of the training phase.

The present review has some limitations. First, the studies included in this review were either single pilot studies or had a cross-over design wherein possible confounding factors were not reported. Second, there were no tools to standardise exercise intensity, and the use of VO_2max_ could be subject to misinterpretation. Third, as serum hepcidin is affected by the circadian rhythm, discrepancies in reported data could be due to the time at which the exercise was performed in the day. There are few reports regarding specific time points of measurement in some studies.

The strength of the present review relies on being the first to investigate the effects of both resistance and endurance training on serum hepcidin response. Moreover, the effects of long-term training on hepcidin, the types of exercise, volume, and duration of exercise regimes, and hepcidin’s responses were also investigated. However, there is a dearth of research on resistance exercise and the association with the regulation of serum hepcidin levels, consequently; further research is, therefore, imperative.

## 5. Conclusions

Iron deficiency is common among athletes and causes deleterious effects on performance due to decreased oxygen delivery and reduced aerobic capacity. Hepcidin plays an important role in the regulation of erythropoiesis and iron absorption. The current review shows that resistance and endurance training both increased serum hepcidin and IL-6 levels in response to exercise. For endurance training, exercise intensities between 60% and 90% VO_2max_ elevated serum hepcidin and IL-6 levels, reaching a peak at 3 h after exercise for hepcidin and immediately after exercise for IL-6. The magnitude of hepcidin response to exercise correlates with the baseline serum ferritin levels and post-exercise serum IL-6. An elevated serum hepcidin level reduces iron absorption and results in reduced erythropoiesis, culminating in iron deficiency and anaemia, which consequently impact exercise performance. 

## Figures and Tables

**Table 1:** PICOS criteria for inclusion and exclusion of studies.

Criteria.	Inclusion	Exclusion
Population	Athletes or recreationally trained subjects	Non-athletes or non-recreationally trained subjects
Intervention/exposure	Physical exercises, either resistance training or aerobic training, at different intensity levels	Exercises that are not defined resistance or aerobic
Comparator	Exercise-induced changes in serum hepcidin and IL-6 levels from baseline	
Outcome	Primary and secondary outcomes are changes in serum hepcidin and IL6 levels from baseline to the last exercise training protocol, respectively.	No evidence of quantitative data or sufficient information to evaluate changes in serum hepcidin
Study design	Experimental studies	Systematic review Meta-analysis Non-experimental studies Conference report

**Table 2:** Characteristics of the studies.

Study (Author/Year)	Design	Country	Sample Size	Sex	Age (Years)	Outcomes	Summary of Results
Goto et al., 2020 [33]	Cross-over design	Japan	10	M	23 + 1	Hepcidin IL-6	There was significantly elevated serum hepcidin after exercise (*p* < 0.001), with significantly greater in the RE (463 ± 125%) than in the endurance training (137 ± 27%, *p* = 0.03). There were significantly increased plasma interleukin-6 (IL-6) levels after exercise by 2.2 pg/mL in resistance training and by 1.6 pg/mL in endurance training (*p* = 0.003), with no significant difference between the trials
Tomczyk et al., 2020 [34]	Single-arm study	Poland	29	M	39.29 ± 8.58	Hepcidin	The hormones that control iron metabolism induced by marathon depend on baseline serum level of iron and ferritin.
McCormick et al., 2019 [35]	Cross-over design	Australia	16 M (10) F (6)	M/F	25.1 ± 4.8	Hepcidin IL-6	There was a significant increase by 0.38 nM for hepcidin and by 4.938 pg/mL for IL-6 concentration. More iron is absorbed at breakfast after morning exercise compared to afternoon exercise.
Dominguez et al., 2020 [31]	Cross-over design	Spain	15	M	31.7 ± 2.6	Hepcidin	There was no significant change in hepcidin level responses to different exercise levels.
Bauer et al., 2018 [36]	Single-arm pilot study	Germany	31 M (19) F (12)	M/F	27.6 ± 7.6 (Males) 27.7 ± 4.5 (Females)	Hepcidin	Significant increase in serum hepcidin by 2.8 ng/mL in men and by 3.7 ng/mL in women.
Goto et al., 2018 [37]	Cross-over design	Japan	10	M	20.9 ± 0.3	Hepcidin IL-6	There was significantly increased serum hepcidin compared to baseline for hypoxic and normal conditions, but there was no significant difference between trials.
Goto et al., 2017 [38]	Cross-over design	Japan	10	M	19.8 ± 0.9	Hepcidin IL-6	Increased serum hepcidin after exercise was observed in both trials
Zugel et al., 2019 [39]	Single-arm study	Germany	8	M	17.8 ± 0.4	Hepcidin	Cumulative training load can affect hepcidin. No change in sTfR levels and ferritin index implied that erythropoiesis was not influenced by iron compartmentalization via hepcidin.
Peeling et al., 2017 [29]	Single-arm pilot study	Australia	24	M	27.2 ± 4.0	Hepcidin IL-6	There was significantly increased serum IL-6 and hepcidin-25 (by 7.5 nM and 8.2 pg/mL, respectively) in response to a 25-km race-walk trial (*p* < 0.05).
Moretti et al., 2018 [40]	Single-arm pilot study	Switzerland	10	M	35 (21–50)	Hepcidin IL-6	A 3-wk exercise resulted in increased serum IL-6, while there was decreased serum hepcidin at the end of the study.
Govus et al., 2017 [41]	Cross-over design	Australia	10 M (6) F (4)	M/F	28.6 ± 6.7	Hepcidin	2 weeks of normobaric hypoxia suppressed resting hepcidin levels from 4 to 2 nM.
Skarpanska et al., 2015 [42]	Single-arm pilot study	Poland	20	M	21.3 ± 0.82	Hepcidin IL-6	There were significant increases in IL-6 by 3.8 pg/mL and in hepcidin by 1.5 nm/mL after exercise. However, there was no significant change in IL-6 and hepcidin in a 24-h recovery period.
Govus et al., 2014 [43]	Cross-over design	Australia	13 M (7) F (6)	M/F	28.8 ± 5.3	Hepcidin IL-6	Hepcidin levels significantly increased at 3 h post-exercise by 0.85 nmol/L in normoxia and by 1.48 nmol in hypoxia, with no significant differences between trials. Significantly increased serum IL-6 immediately post-exercise by 0.61 ng/mL in normoxia and by 0.68 ng/mL, but returned to baseline 3 h later.
Sim et al., 2014 [44]	Cross-over design	Australia	10	M	24 ± 1	Urinary hepcidin level	There was a significantly increased urinary hepcidin level (*p* ≤ 0.05).
Peeling et al., 2014 [45]	Single-arm pilot study	Australia	54 M (38) F (16)	M/F	25.8 ± 6.6	Hepcidin IL-6	Significantly increased serum IL-6 after exercise was observed when compared to baseline within each group (*p* < 0.05). Post-exercise hepcidin elevation was observed when the baseline serum ferritin was above 30.
Badenhorst et al., 2014 [46]	Cross-over design	Australia	10	M	26.6 ± 10.7	Hepcidin IL-6	Significantly increased serum IL-6 immediately after exercise compared to baseline level, but there was no difference between conditions. There was significantly elevated serum hepcidin at 3 h post-exercise for both trials compared to baseline, but it was significantly lower in the hypoxic condition.
Antosiewicz et al., 2013 [47]	Single-arm pilot study	Poland	21	M	22 ± 1.5	Hepcidin IL-6	There was significantly increased serum IL-6 and hepcidin at 1 h after exercise in both groups (*p* < 0.05). Hepcidin returned to baseline level in 24 h in judo athletes, whereas in control group, it remained increased for 5 days following exercise.
Auersperher et al., 2013 [48]	Single-arm pilot study	Slovenia	14	F	31.4 ± 5.9 for group that had ferritin levels > 20 mg/L 34.9 ± 4.7 for group that had ferritin levels < 20 mg/L	Hepcidin IL-6	There was no significant difference in serum IL-6 and hepcidin levels during training and baseline. However, hepcidin was significantly lower at recovery compared with baseline (*p* < 0.05).
Sim et al., 2013 [25]	Cross-over design	Australia	10	M	23 ± 1	Hepcidin IL-6	Significant increases in post-exercise IL-6 level were seen within each trial (*p* < 0.05) and were significantly greater in H-R (by 6 pg/mL) than L-R (*p* < 0.05). There was a significant increase in serum hepcidin levels at 3 h post-exercise within each trial (*p* < 0.05).
Newlin et al., 2012 [26]	Cross-over design	USA	12	F	19–32	Hepcidin IL-6	Hepcidin significantly increased at 3 h post-exercise then began to decline by 6 and 9 h post-exercise. IL-6 was significantly increased immediately post-exercise.
Auersperger et al., 2012 [49]	Single pilot study	Slovenia	18	F	Interval group (*N* = 10) 32.9 ± 5.7 years Continuous group (*N* = 8) 31.6 ± 4.8 years	Hepcidin IL-6	There was decreased serum hepcidin with time in TPost1 and in BPost compared with BPre (*p* < 0.001) and increased in TPost2 compared with TPost1 (*p* < 0.001). IL-6 concentrations were not detected at all time points due to the plasma concentrations being below 2 ng/L.
Kasprowicz et al., 2013 [50]	Single-arm pilot study	Poland	6	M	44.5 ± 13.5	Hepcidin IL-6	A 100-km run caused a progressive increase in blood IL-6 concentration, which reached the highest values after 75 km. The 100-km race did not affect blood hepcidin concentration.
Skarpanska-Stejnborn et al., 2019 [51]	Single pilot study	Poland	15	M	21 ± 1	Hepcidin IL-6	There were significant increases in IL-6 by 4 pg/mL and in hepcidin by 0.2 ng/mL. The level of hepcidin and IL-6 dropped to baseline after a 1-day recovery.

M = Male; F = Female., H-R = High-intensity interval run session at 85% VO2max; L-R = Low-intensity continuous run at 65% VO2max; TPost1 = progressive training load at week 1–3; BPost = Final laboratory test at week 10; BPre = Laboratory tests at baseline; TPost2 = progressive training load at week 5–7.

**Table 3:** Summary of the studies evaluating the effect of exercise on serum hepcidin and IL-6 concentrations.

Author	Population	Sample Size	Exercise Protocol	Time Points Measurement	Main Outcomes Pre vs. Post
Goto et al., 2020 [33]	Recreationally trained males	10	EP1: RT at 60% 1RM, 3–5 sets × 12 reps EP2: END, 65% VO_2max_ × 60 min EP3: Rest trial: reading books and watching DVDs	Pre, 1 h, 2 h, 3 h, and 6 h PE	Hepcidin (Pre, 3 h, and 6 h PE) (ng/mL): EP1: ~8.0 vs. ~42 * vs. ~26 * EP2: ~14 vs. ~32.5 * vs. ~20 IL-6 (Pre, 0 h, 1 h, and 6 h PE) (pg/mL): EP1: ~1.0 vs. ~3.9 * vs. ~4.1 * vs. ~3.5 EP2: ~1.2 vs. ~4.2 * vs. ~2.9 vs. ~2.7 EP3: Pre and 3 h PE Hepcidin: ~6 vs. ~9 nm/mL Il-6: ~1 vs. ~1.1 pg/ml
Tomczyk et al., 2020 [34]	Marathon runners	29	Marathon Long-distance continuous running	Pre (1 month before the marathon) Post-1 sample (after the marathon) Post-2 samples (39 ± 2 days after the competition)	Hepcidin (ng/mL) 1.12 vs. 1.09 * vs. 0.92
McCormick et al., 2019 [35]	Endurance-trained runner Males	16	EP1: Running protocol (65% VO_2max_) in the morning EP2: running protocol (65% VO_2max_) in the afternoon	Pre, 0 h, and 3 h PE	Hepcidin (Pre and 3 h PE) (ng/mL) EP1: 1.11 vs. 6.69 * EP2: 5.02 vs. 13.38 * IL-6 (Pre and 3 h PE) (pg/mL) EP1: 1 vs. 5.5 * EP2: 6 vs. 9 Fractional iron absorption (breakfast vs. dinner) EP1: 8% vs. 7.5% EP2: 7% vs. 6.9%
Domínguez et al., 2020 [31]	Trained cyclists	15	EP1: 30-min moderate-intensity continuous cycling EP2: 30-min high-intensity continuous cycling	Pre- and Post-exercise	Hepcidin(ng/mL) EP1: 1.28 vs. 1.29 EP2: 1.28 vs. 1.24 Norepinephrine (pg/mL) EP1: 366.22 vs. 794.4 EP2: 366.22 vs. 1622
Bauer et al., 2018 [36]	Elite	31	High-intensity exercise with 70–80% VO_2max_ and up to 90–100% VO_2max_ in sprint distances 200–1000 m	Pre and 3 h PE	Hepcidin (Pre- and Post-exercise) (ng/mL) 8.7 vs. 12 * Iron (Pre- and Post-exercise) (µg/dL) 105.9 vs. 63.5 *
Goto et al., 2018 [37]	Athletes	10	EP1: exercise trials under normoxic conditions (FiO_2_: 20.9%) EP2: exercise trials under hypoxic conditions (FiO_2_: 14.5%)	Pre, 0 h, 1 h, and 3 h PE	Hepcidin (Pre, 0 h, 1 h, and 3 h PE) (ng/mL) EP1: 16 vs. 18 vs. 19 vs. 30 * EP2: 11 vs. 13 vs. 10 vs. 19 * IL-6 (Pre, 0 h, 1 h, and 3 h PE) (pg/mL) EP1: 1.3 vs. 2.7 * vs. 2.5 * vs. 1.2 EP2: 0.8 vs. 1.8 * vs. 2.2 * vs. 1.0
Goto et al., 2017 [38]	Endurance athletes	10	EP1: HIT in endurance exercise under hypoxic condition (FiO_2_ = 14.5%) EP2: HIT in endurance exercise under a normoxic condition (FiO_2_ = 20.9%)	Pre, 0 h, 1 h, and 2 h PE	Hepcidin (ng/mL) EP1: 10.7 vs. 12.7 vs. 11.8 vs. 15.8 * EP2: 7.9 vs. 10.1 vs. 9.7 vs. 13.2 * IL-6 (pg/mL) EP1: 0.6 vs. 5.2 * vs. 2.9 * vs. 2.0 * EP2: 0.50 vs. 5.9 * vs. 3.4 * vs. 2.0 *
Zügel et al., 2020 [39]	Junior world elite rowers	8	A 4-week endurance training camp EP1:D0: TV:104 km/wk (low training load) EP2:D7: TV:180 km/wk (high training load) EP3:D13:TV:202 km/wk (high training load) EP4:D18:TV:170 km/wk (high training load) EP5:D24:TV:133 km/wk (moderate training load) EP6:D28:TV 133 km/wk (moderate training load)	Pre- and post-exercise training at D 0, 7, 13, 18, 24, and 28	Hepcidin EP1: ~11.5 ng/mL EP2: ~23.2 * ng/mL EP3: ~9.5 ng/mL EP4: ~10.7 ng/mL EP5: ~7.2 ng/mL EP6: ~7.5 ng/mL Iron EP1: ~12 µmol/l EP2: ~17 µmol/l EP3: ~18 µmol/l EP4: ~16 µmol/l EP5: ~19 * µmol/l EP6: ~15 µmol/l
Peeling et al., 2017 [29]	Male race walkers	24	25-km race-walk trial Intensity: 75% of VO_2max_	Pre and 3 h PE	Hepcidin (Pre and 3 h PE) (ng/mL) 3.0 vs. 23.9. * IL-6 (Pre and 3 h PE) (pg/mL) 1.2 vs. 9.4 * Correlation 3 h post-exercise hepcidin was significantly correlated to baseline serum ferritin (R = 0.69) and baseline serum iron (R = 0.62).
Moretti et al., 2018 [40]	Recreation male runners	10	EP1: control phase, maintain usual intensity for 16 days from D 1 to D 16 EP2: Exercise phase, ran 8 km every second day (80% HR max) for a total of 11 training sessions from D17 to 29	D 1, 7, 22, and 29	Hepcidin (D 7 and 29) (ng/mL) EP1 vs. EP2 20.2 vs. 14.4 * ng/mL IL-6 (D 7 and 29) (pg/mL) EP1 vs. EP2 0.87 vs. 5.17 * Iron absorption (D 7 and 29) EP1 vs. EP2 15.6 vs. 19.3% EPO (D 7 and 29) (IU/L) EP1 vs. EP2 0.66 vs. 2.06 *
Govus et al., 2017 [41]	Well-trained middle- or long-distance runners	10	EP1: HIT in endurance exercise under hypoxic condition (FiO_2_ = 15.5%) EP2: HIT in endurance exercise under a normoxic condition (~600 m natural altitude). EP3: 11 days of LHTL	Pre and 3 h PE for EP1 and EP2 Pre and 2 weeks PE for LHTL	Hepcidin (ng/mL) EP1 (Pre vs. 3 h PE) 11.4 vs. 18.1 * EP2: (Pre vs. 3 h PE) 10.3 vs. 20.6 * EP3 (Pre vs. 2 weeks PE) 11.1 vs. 5.5 *
Skarpańska-Stejnborn et al., 2015 [42]	Male rowing athletes	20	Endurance exercise 2000 m (high intensity) maximal test	Pre, 0 h and 1 D PE	Hepcidin (ng/mL) 0.25 vs.1.7 * vs. 0.25 IL-6 (pg/mL) 1.8 vs. 5.5 * vs. 1.5 Iron (mg/dL) 144 vs. 156 vs. 110
Govus et al., 2014 [43]	Endurance athletes	13	EP1: HIT in endurance exercise under hypoxic condition (FiO_2_ = 14.5%) EP2: HIT in endurance exercise under a normoxic condition (FiO_2_ = 20.9%)	Pre, 0 h and 3 h PE	Hepcidin (ng/mL) Pre vs. 3 h PE EC1: 0.9 vs. 1.4 * EC2: 1.0 vs. 1.3 * IL-6 (pg/mL) Pre vs. 3 h PE EC1: 560 vs. 1240 * EC2: 500 vs. 1100 *
Sim et al., 2014 [44]	Trained males	10	EP1: running with 78–89% max HR EP2: cycling with 78–89% max HR	Pre and 3 h PE at D 1, 2, and 6	Urinary hepcidin (nM/mmol Cr) EP1 D1: 0.46 vs. 1.19 * D2: 0.76 vs. 1.38 D6: 0.71 vs. 0.93 EP2 D1: 0.52 vs. 0.64 * D2: 0.85 vs. 0.84 D6: 0.43 vs. 0.80
Peeling et al., 2014 [45]	Trained runners or triathletes	54	EC1. Endurance exercise with serum ferritin < 30 mg/L EC2. Endurance exercise with serum ferritin 30–50 mg/L EC3. Endurance exercise with serum ferritin 50–100 mg/L EC4. Endurance exercise with serum ferritin >100 mg/L	Pre and 3 h PE	Hepcidin (ng/mL) EC1: ~2.5 vs. ~3.3 EC 2: ~5.8 vs. ~12.5 * EC 3: ~6.1 vs. ~14.7 * EC 4: ~9.4 vs. ~22.5 * IL-6 (pg/mL) EC 1: 1.3 vs. 3.8 * EC 2: 1.1 vs. 3.5 * EC 3: 1.7 vs. 4.3 * EC 4: 1.1 vs. 5.6 * Correlation A significant moderate positive correlation between serum hepcidin and serum ferritin (R = 0.52, *p* < 0.05)
Badenhorst et al., 2014 [46]	Well-trained male endurance athletes	10	Two 8 × 3 min interval running sessions at 85% VO_2max_	Pre, 3 h and 1 D (Hepcidin) Pre and 0 h PE (IL-6)	Hepcidin (ng/mL) EC1: 8.9 vs. 15.1 * vs. 8.7 EC2: 9.0 vs. 20.6 * vs. 11.7 IL-6 (pg/mL) EC1: 1.32 vs. 2.99 * EC2: 1.08 vs. 3.12 *
Antosiewicz et al., 2013 [47]	Male judo athletes	21	Endurance exercise with high-intensity 3 × 30 s all-out sprint (cycle)	Pre, 1 h, 24 h, and 5 D PE	Hepcidin (ng/mL) EC1: 64 vs. 83 * vs. 66 vs. 75 IL-6 (pg/mL) EC1: 1.25 vs. 2.2 * vs. 1.0 vs. 1.8
Auersperger et al., 2013 [48]	Runners	14	Endurance exercise consists of one or two interval trainings: -One at 88–95% MHR, the second up to 100% MHR; -Two easy runs (at 70–87% MHR) of 6–8 and 12–18 km.	Pre, 1 D, and 10 D PE	Hepcidin (ng/mL) SF1: 190 vs. 203 vs. 92.6 * SF2: 173 vs. 134 vs. 102 * IL-6 levels were below a detectable plasma concentration
Sim et al., 2013 [25]	Well-trained male triathletes	10	EP1: 40-min low-intensity continuous run at 65% of VO_2max_ (L-R) EP2: 40-min high-intensity interval run session at 85% of VO_2max_ (H-R) EP3: 40-min low-intensity continuous cycle at 65% of VO_2max_ (L-C) EP4: 40-min high-intensity interval cycle session at 85% of VO_2max_ (H-C)	Pre, 0 h, and 3 h PE	Hepcidin (ng/mL) Pre vs. 3 h PE EP1: ~0.5 vs. ~0.7 * EP2: ~0.5 vs. ~0.7 * EP3: ~0.3 vs. ~0.5 * EP4: ~0.3 vs. ~0.7 * IL-6 (pg/mL) Pre vs. 0 h PE EP1: ~3.4 vs. ~4.5 * EP2: ~3.5 vs. ~6.0 * EP3: ~2.7 vs. ~4.9 * EP4: ~3.1 vs. ~5.4 *
Newlin et al., 2012 [26]	Runners	12	EP1: 60-min endurance exercise by treadmill running at 65% of VO_2max_. EP2: 120-min endurance exercise by treadmill running at 65% of VO_2max_.	Pre, 0 h, 3 h, 6 h, 9 h, and 24 h P	Hepcidin (ng/mL) EP1: 0.23 vs. 0.25 vs. 0.57 * vs. 0.37 vs. 0.28 vs. 0.23 EP2: 0.31 vs. 0.34 vs. 1.38 * vs. 1.00 vs. 0.60 vs. 0.43 IL-6 (pg/mL) EP1: 1.5 vs. 2.9 * vs. 1.9 vs. 1.6 vs. 1.8 vs. 1.4
Auersperger et al., 2012 [49]	Trained female runners	18	EP1: Interval training exercise comprised of two interval training (one at 88–95% MHR, second up to 100% MHR) and two easy runs at 70–87% EP2: Continuous-training exercise comprised of one easy interval training (80–80% MHR) and two easy runs (at 70–87% MHR)	Pre (wk0) TPost1 (wk 3) TPost2 (wk 7) Recovery1 (wk 4) Recovery2 (wk 8) Post-study (wk 10).	Hepcidin (ng/mL) EP1: 170 vs. 105 * vs. 170 vs. 190 vs. 180 vs. 115 * EP2: 180 vs. 80 * vs. 140 vs. 135 vs. 125 vs. 90 *
Kasprowicz et al., 2013 [50]	Male ultra-marathon runners	6	Endurance exercise (running) 100 km	Pre, 25 km, 50 km, 75 km, 100 km, and 14 h PE	Hepcidin (ng/mL) ~43 vs. ~44 vs. ~45 vs. ~42 vs. ~44 vs. ~47 IL-6 (pg/mL) ~1.9 vs. ~7.8 * vs. ~12.5 * vs. ~14 * vs. ~13 * vs. ~5
Skarpańska-Stejnborn et al., 2019 [51]	Elite rowers	15	EP1: Endurance training during the preparatory phase (trial I) EP2: Endurance training during the competition phase (trial II) (Total volumes were higher than EP1)	Pre, 0 h, and 1 D PE	Hepcidin (ng/mL) Ep1: 0.38 vs. 0.50 * vs. 0.36 EP2: 0.38 vs. 0.85 * vs. 0.4 IL-6 (pg/mL) EP1: 2 vs. 6 * vs. 1.9 EP2: 2.1 vs. 7.0 * vs. 1.8

* *p* for trend values are in line with the results to which they are relevant unless otherwise stated. Abbreviations: D = day; RT = resistance training; RM = repetition maximum; ENT = endurance training; PE = post-exercise; HR = heart rate; MHR = maximum heart rate; TV = total volume; HIT = high-intensity interval training; EC = experiment condition; EP = exercise protocol; F_I_O_2_ = fraction of inspired oxygen; LHTL = live high, train low; min = minutes; h = hour; wk = week; NR = not reported; SF = serum ferritin; VO_2max_ = maximum oxygen uptake; ~ = estimated from the figures provided by authors; * significantly different from baseline levels.

## Data Availability

Data are contained in this article and supplementary material.

## Supplementary Materials

The following are available online at https://www.mdpi.com/2072-6643/13/2/393/s1. Supplementary Data: Risk of bias summary: review authors’ judgements about each risk of bias item for each included study.

## Abbreviations

AAE	Acute anaemia of exercise
BMP	Bone morphogenetic protein
DMT1	Divalent metal transporter 1
EPO	Erythropoietin
ERFE	Erythroferrone
Fe^2+^	Ferrous iron
Fe^3+^	Ferric iron
FPN	Ferroportin
HAMP	Hepcidin antimicrobial peptide
HIF	Hypoxia-inducible factor
ID	Iron deficiency
IL-6	Interleukin-6
JAK/STAT3	The Janus kinase (JAK)-signal transducer and activator of transcription (STAT) 3
LSECs	Liver sinusoidal endothelial cells
PICOS	Population, Intervention, Comparison, Outcomes and Study (PICOS)
TfR2	Transferrin receptor 2
TIBC	Total iron-binding capacity
VHL	von Hippel–Lindau factor
VO_2max_	Maximal oxygen uptake
CRP	C-reactive protein
sTfR	Soluble transferrin receptor
