# Supplementary material for: Association of Serum Hepcidin Levels with Aerobic and Resistance Exercise: A Systematic Review
Source: Nutrients. 2021 Jan 27;13(2):393. doi: 10.3390/nu13020393 (PMC7911648; doi:10.3390/nu13020393)
Supplement: Supplementary file 1 [file nutrients-13-00393-s001.pdf]

Supplementary : Risk of bias summary: review authors' judgements about each risk of bias item for each included study.

1. Was the study question or objective clearly stated?

2. Were eligibility/selection criteria for the study population prespecified and clearly described?

3. Were the participants in the study representative of those who would be eligible for the test/service/intervention in the general or clinical population of interest?

4. Were all eligible participants that met the prespecified entry criteria enrolled?

5. Was the sample size sufficiently large to provide confidence in the findings?

6. Was the test/service/intervention clearly described and delivered consistently across the study population?

7. Were the outcome measures prespecified, clearly defined, valid, reliable, and assessed consistently across all study participants?

8. Were the people assessing the outcomes blinded to the participants' exposures/interventions?

9. Was the loss to follow-up after baseline 20% or less? Were those lost to follow-up accounted for in the analysis?

10. Did the statistical methods examine changes in outcome measures from before to after the intervention? Were statistical tests done that provided p values for the pre-to-post changes?

11. Were outcome measures of interest taken multiple times before the intervention and multiple times after the intervention (i.e., did they use an interrupted time-series design)?

12. If the intervention was conducted at a group level (e.g., a whole hospital, a community, etc.) did the statistical analysis take into account the use of individual-level data to determine effects at the group level?

Quality rating

Goto et al 2020	Yes	Yes	No	NR	NR	Yes	Yes	NR	Yes	Yes	NR	NA	Fair
Tomczyk et al 2020	Yes	CD	No	NR	NR	CD	Yes	NR	No	Yes	NR	NA	Poor
McCormick et al 2019	Yes	Yes	Yes	NR	NR	Yes	CD	NR	Yes	Yes	NR	NA	Fair
Dominguez et al 2020	Yes	Yes	No	NR	NR	Yes	Yes	NR	Yes	Yes	NR	NA	Poor
Bauer *et* al 2018	Yes	Yes	Yes	NR	NR	Yes	Yes	NR	Yes	Yes	NR	NA	Fair
Goto et al 2018	Yes	Yes	No	NR	NR	Yes	Yes	NR	Yes	Yes	NR	NA	Fair
Goto el al 2017	Yes	Yes	No	NR	NR	Yes	Yes	NR	Yes	Yes	NR	NA	Fair
Zugel et al 2019	Yes	CD	No	NR	NR	Yes	Yes	NR	Yes	No	NR	NA	Poor
Peeling et al 2017	Yes	Yes	No	NR	NR	Yes	Yes	NR	Yes	Yes	NR	NA	Fair

Moretti et al 2018	Yes	Yes	No	NR	Yes	Yes	Yes	NR	Yes	Yes	NR	NA	Good
Govus et al 2016	Yes	Yes	Yes	NR	NR	Yes	No	NR	Yes	Yes	NR	NA	Poor
Skarpanska et al 2015	Yes	Yes	No	NR	NR	Yes	Yes	NR	Yes	Yes	NR	NA	Fair
Govus et al 2014	Yes	CD	Yes	NR	NR	Yes	No	NR	Yes	Yes	NR	NA	Poor
Sim et al 2014	Yes	Yes	No	NR	Yes	Yes	Yes	NR	Yes	Yes	NR	NA	Good
Peeling et al 2014	Yes	Yes	Yes	NR	NR	Yes	Yes	NR	Yes	Yes	NR	NA	Fair
Badenhorst et al 2014	Yes	Yes	No	NR	Yes	Yes	Yes	NR	Yes	Yes	NR	NA	Good
Antosiewicz et al 2013	Yes	Yes	No	NR	NR	Yes	Yes	NR	Yes	Yes	NR	NA	Fair
Auersperher et al 2013	Yes	Yes	No	NR	NR	Yes	Yes	NR	Yes	Yes	NR	NA	Fair
Sim et al 2013	Yes	Yes	No	NR	Yes	Yes	Yes	NR	Yes	Yes	NR	NA	Good
Newlin et al 2012	Yes	Yes	No	NR	Yes	Yes	Yes	NR	Yes	Yes	NR	NA	Good

Auersperger et al 2012	Yes	Yes	No	NR	NR	Yes	Yes	NR	Yes	Yes	NR	NA	Fair
Kasprowicz et al 2013	Yes	Yes	No	NR	NR	No	Yes	NR	Yes	Yes	NR	NA	Poor
Skarpanska-Stejnborn et al 2019	Yes	Yes	No	NR	NR	CD	Yes	NR	Yes	Yes	NR	NA	Fair

Appendix C: Risk of bias graph: review authors' judgements about each risk of bias item presented as percentages across all included studies.

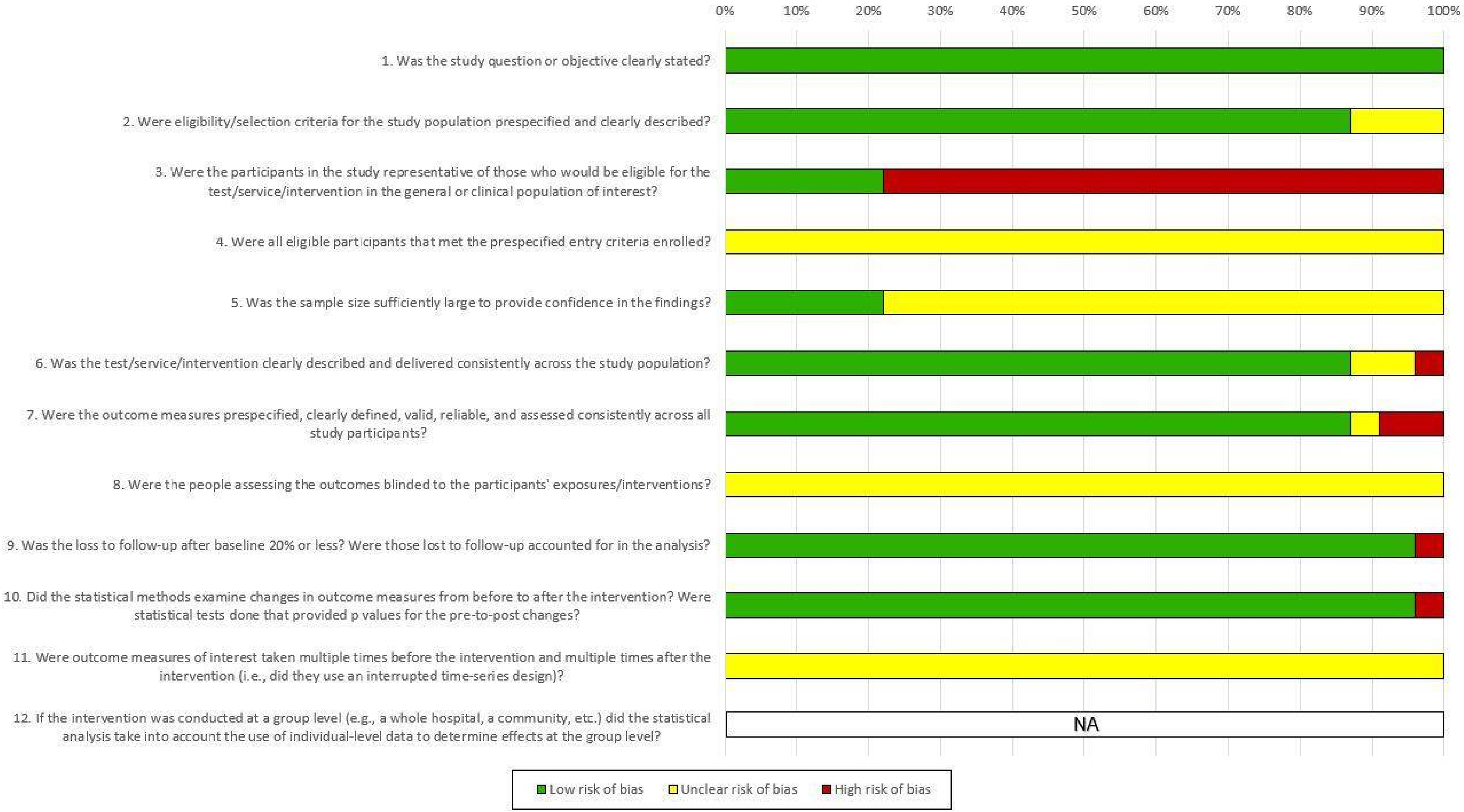
0%
10%
20%
30%
40%
50%
60%
70%
80%
90%
100%
1. Was the study question or objective clearly stated?
2. Were eligibility/selection criteria for the study population prespecified and clearly described?
3. Were the participants in the study representative of those who would be eligible for the test/service/intervention in the general or clinical population of interest?
4. Were all eligible participants that met the prespecified entry criteria enrolled?
5. Was the sample size sufficiently large to provide confidence in the findings?
6. Was the test/service/intervention clearly described and delivered consistently across the study population?
7. Were the outcome measures prespecified, clearly defined, valid, reliable, and assessed consistently across all study participants?
8. Were the people assessing the outcomes blinded to the participants' exposures/interventions?
9. Was the loss to follow-up after baseline 20% or less? Were those lost to follow-up accounted for in the analysis?
10. Did the statistical methods examine changes in outcome measures from before to after the intervention? Were statistical tests done that provided p values for the pre-to-post changes?
11. Were outcome measures of interest taken multiple times before the intervention and multiple times after the intervention (i.e., did they use an interrupted time-series design)?
12. If the intervention was conducted at a group level (e.g., a whole hospital, a community, etc.) did the statistical analysis take into account the use of individual-level data to determine effects at the group level?
NA
Low risk of bias
Unclear risk of bias
High risk of bias
